# Whole Body Vibration: A Valid Alternative Strategy to Exercise?

**DOI:** 10.3390/jfmk7040099

**Published:** 2022-11-03

**Authors:** Roberto Bonanni, Ida Cariati, Cristian Romagnoli, Giovanna D’Arcangelo, Giuseppe Annino, Virginia Tancredi

**Affiliations:** 1 Department of Clinical Sciences and Translational Medicine, “Tor Vergata” University of Rome, Via Montpellier 1, 00133 Rome, Italy; 2 Sport Engineering Lab, Department of Industrial Engineering, “Tor Vergata” University of Rome, Via Politecnico 1, 00133 Rome, Italy; 3 Department of Systems Medicine, “Tor Vergata” University of Rome, Via Montpellier 1, 00133 Rome, Italy; 4 Centre of Space Bio-Medicine, “Tor Vergata” University of Rome, Via Montpellier 1, 00133 Rome, Italy

**Keywords:** whole-body vibration, physiological adaptations, cognitive function, neurodegeneration, musculoskeletal disorders, pain, exercise, prevention, alternative strategy

## Abstract

Several studies agree that mechanical vibration can induce physiological changes at different levels, improving neuromuscular function through postural control strategies, muscle tuning mechanisms and tonic vibration reflexes. Whole-body vibration has also been reported to increase bone mineral density and muscle mass and strength, as well as to relieve pain and modulate proprioceptive function in patients with osteoarthritis or lower back pain. Furthermore, vibratory training was found to be an effective strategy for improving the physical performance of healthy athletes in terms of muscle strength, agility, flexibility, and vertical jump height. Notably, several benefits have also been observed at the brain level, proving to be an important factor in protecting and/or preventing the development of age-related cognitive disorders. Although research in this field is still debated, certain molecular mechanisms responsible for the response to whole-body vibration also appear to be involved in physiological adaptations to exercise, suggesting the possibility of using it as an alternative or reinforcing strategy to canonical training. Understanding these mechanisms is crucial for the development of whole body vibration protocols appropriately designed based on individual needs to optimize these effects. Therefore, we performed a narrative review of the literature, consulting the bibliographic databases MEDLINE and Google Scholar, to i) summarize the most recent scientific evidence on the effects of whole-body vibration and the molecular mechanisms proposed so far to provide a useful state of the art and ii) assess the potential of whole-body vibration as a form of passive training in place of or in association with exercise.

## 1. Introduction

Scientific research on the effects of mechanical vibrations has clearly highlighted the danger that this stimulus can pose to the state of health, emphasizing how the risk from vibrations is generated when using specific tools, working instruments and machinery that induce continuous stresses in the body of the worker using them, compromising apparatuses, joints, or even internal organs [1,2]. Furthermore, the study of vibrations’ effect on the human body has led, in recent years, to a greater understanding of degenerative phenomena leading to a broad spectrum of occupational diseases. However, there are still poorly documented aspects of both foot-transmitted vibration (FTV) and hand–arm vibration (HAV) [3,4].

A further condition is the whole-body vibration (WBV) to which many workers are exposed while driving trucks and agricultural machinery, or through the use of tools that produce high-amplitude vibrations that are transferred to the entire body, damaging various organs and apparatuses [5,6]. In this context, prolonged exposure to vibration in the construction work environment has been reported to be significantly associated with musculoskeletal disorders, predominantly in the neck, shoulder, and arm [7,8]. Notably, such disorders induced by WBV exposure are manifested by musculoskeletal pain, the chronicity of which, due to the persistence of the stimulus, leads to reduced hours of work activity, impaired emotional well-being and, in general, a worsening of the individual’s quality of life [9,10].

Although a negative impact of vibrations has also been documented in the peripheral nervous system, the digestive system, the female reproductive system, and the vestibular system [11,12,13,14], an increasing part of the literature reports scientific evidence in favor of the use of WBV as a form of alternative training in patients unable to exercise [15]. In this context, the beneficial effects of WBV appear to be numerous, including the prevention of chronic and degenerative diseases. Vibratory stimulation has also been suggested as an effective tool to prevent and/or counteract age-related cognitive decline and to mitigate the physiological changes that characterize aging [16]. Importantly, the utility of WBV does not appear to be limited to the prevention of disease and aging, but is also often applied for rehabilitation purposes in athletes with various conditions, including to improve balance in individuals with ankle instability [17], to increase muscle strength in individuals with anterior cruciate ligament reconstruction [18], and to reduce patellofemoral pain by optimizing sports performance [19].

Although WBV represents a powerful stimulus for the entire organism, the underlying biological mechanisms have not yet been fully elucidated, due in part to the extreme variability of its effects, which are strongly dependent upon the parameters that characterize mechanical vibration, such as the frequency and amplitude of the vibration as well as the duration of vibration exposure [20]. Nevertheless, a complete understanding of the physiological adaptations to WBV and the consequent application of protocols customized to individual needs should be the primary goal of research in this field, providing a valid strategy to counteract the progression of various degenerative diseases, as well as age-related physiological decline in individuals unable to exercise. Furthermore, depending on the needs of individual athletes, specific WBV protocols, sometimes administered at the same time as exercise, could prove extremely useful in the rehabilitation of musculoskeletal disorders as well as for improving sports performance.

Therefore, the aim of our review was to (i) summarize the scientific information and experimental data on physiological adaptations to WBV with a focus on the effects on cognitive function, musculoskeletal health, and pain perception, and (ii) gather evidence on the clinical efficacy of WBV in order to consider its use as an alternative strategy to exercise.

## 2. Literature Search Strategy

A non-systematic search strategy was adopted for the writing of this narrative review, selecting 100 scientific articles concerning the effects of vibratory training on the entire organism, with a focus on nervous and musculoskeletal tissues. The bibliographic databases MEDLINE and Google Scholar were used to select peer-reviewed articles of interest published between 1945 (start date) and 2022. The search strategy was based on the use of the following combinations of medical subject headings (MeHS) and keywords: (whole body vibration) AND (benefits); (whole body vibration) AND (nervous system); (whole body vibration) AND (cognitive function); (whole body vibration) AND (neurodegeneration); (whole body vibration) AND (brain); (whole body vibration) AND (musculoskeletal system); (whole body vibration) AND (bone); (whole body vibration) AND (muscle); (whole body vibration) AND (osteoporosis); (whole body vibration) AND (osteoarthritis); (whole body vibration) AND (sarcopenia); (whole body vibration) AND (pain); (whole body vibration) AND (low back pain); (whole body vibration) AND (fibromyalgia); (whole body vibration) OR (prevention); (whole body vibration) AND (exercise); (whole body vibration) OR (alternative strategy). For each combination listed, the keyword “whole body vibration” was replaced with the terms “vibratory training”, “mechanical vibration” and “WBV”. The results included in vitro and in vivo experimental studies, systematic reviews and meta-analyses, narrative reviews, randomized controlled trials and clinical trials to provide a comprehensive overview.

All search results were analyzed by two researchers who defined their relevance to the topic. Any disagreements during the article selection process were resolved through discussion with a third researcher. Finally, a further check of the reference lists was conducted by two other authors who confirmed the validity of the search performed and clarified any doubts. The search process was performed on a worldwide basis, without excluding specific geographical areas or different ethnic groups. Language and species filters were applied to the list of results to eliminate non-English language articles.

## 3. Physiological Adaptations to WBV

**Figure 1:**
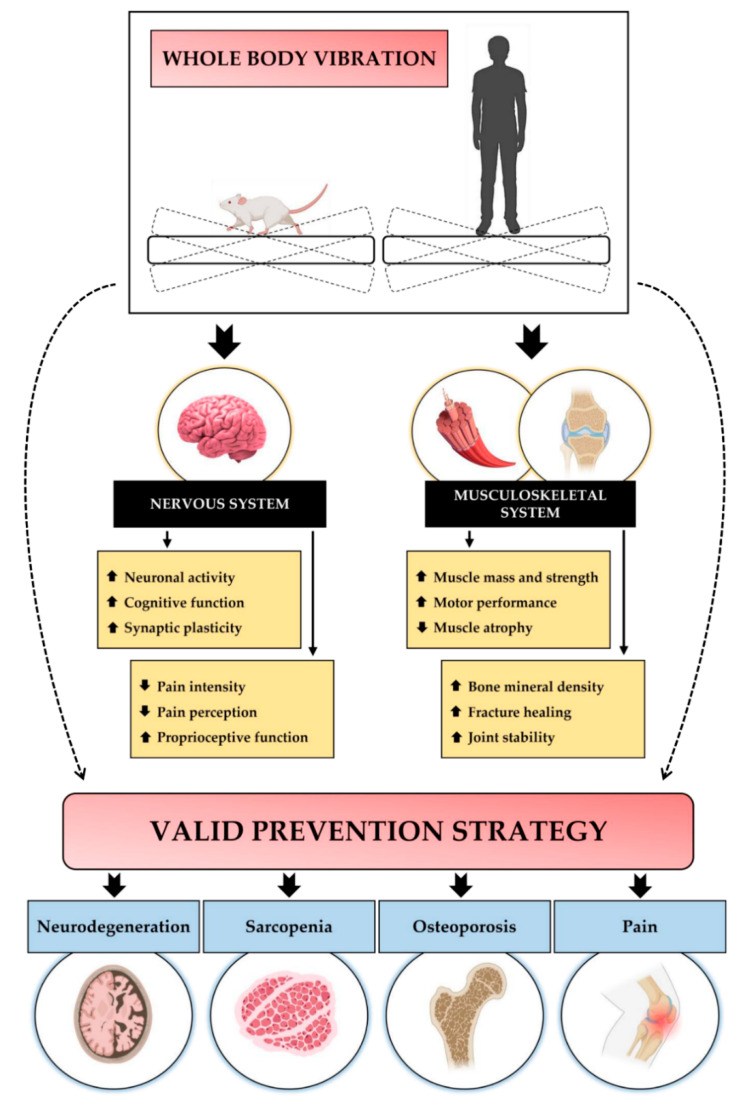
A schematic representation of the WBV effects on the nervous and musculoskeletal systems. WBV improves brain health by increasing neuronal activity, cognitive function, and synaptic plasticity. At the nervous system level, WBV also promotes proprioceptive function, reducing the intensity and perception of pain. At the level of the musculoskeletal system, WBV increases muscle mass and strength, as well as motor performance, while reducing muscle atrophy. In addition, vibratory training increases bone mineral density and promotes fracture healing and joint stability. Taken together, these effects make WBV a valuable preventive strategy for neurodegenerative diseases, musculoskeletal disorders such as sarcopenia and osteoporosis, and pain-associated diseases.

Most of the knowledge regarding the molecular mechanisms underlying physiological adaptations to WBV is provided by studies conducted on rodents, which have shown that the positive effects of vibratory training depend on specific parameters, such as vibration frequency, and vibration exposure time. In particular, most studies agree that the main benefits are associated with low-intensity WBV protocols, with variable effects mainly affecting nervous and musculoskeletal tissue [21,22,23] (Figure 1).

### 3.1. WBV Improves Cognitive Function and Counteracts Neurodegeneration

The transmission of vibrations and oscillations to the body is known to stimulate skin receptors, muscle spindles and the vestibular system, inducing numerous changes in brain activity, such as those in the somatosensory cortex, thalamus, hippocampus, and amygdala, and altering the concentrations of important neurotransmitters, such as dopamine and serotonin, which act as messengers for nervous cells [24,25,26]. The positive effects of WBV on brain function were mainly observed through studies in animal models, which showed that 5-week WBV training increased the activity of the cholinergic system in the somatosensory cortex and amygdala of C57Bl/6J mice [27], and preliminary studies found a WBV-induced enhancement of immediate and early c-fos gene expression, indicative of increased neuronal activity [28]. In agreement with this, a significant improvement in neurological and motor abilities was found in female rats exposed to a low-frequency WBV protocol for 30 days, in association with a reduction in post-stroke inflammation and frailty that prevented post-ischemic cognitive decline. [29]. More recently, Peng and colleagues studied the effects of 8 weeks of vibration training on neuronal loss, synaptic protein expression and neurotrophic factor levels in a rat model with chronic restraint stress-induced depression (CRS). Interestingly, a significant improvement in cognitive function was observed, in addition to neuroprotection and reduction of neuronal damage and death, suggesting WBV as a powerful therapeutic strategy for major depressive disorder [30]. Similarly, Cariati et al. investigated changes in synaptic plasticity by means of electrophysiological recordings of the hippocampus in 4-month-old and 24-month-old young mice exposed to different WBV protocols, identifying the protocol characterized by a lower vibration frequency and longer recovery time as the only one capable of positively modulating hippocampal synaptic plasticity and influencing the higher cognitive processes of learning and memory. In contrast, the other vibration training protocols, which differed in vibration frequency, vibration exposure time and recovery time, were found to be too stressful, inducing the appearance of an epileptic tendency and possibly damaging the hippocampus and other brain structures related to memory functions [31]. These results were later confirmed by the same authors through histological and morphometric analyses, showing that reduced synaptic function was associated with the presence of structural alterations, including a reduction in the number of Purkinje cells in the cerebellum, as well as reduced or even absent pyramidal neurons in the hippocampus, indicating WBV-induced sensory stimulation as an essential part of the mechanism underlying the improved cognitive performance in mice [32].

Brain adaptations to WBV could also involve multiple brain regions in humans, given the close communication between the sensory systems of vibration perception and the areas that oversee cognitive functions [33]. In this regard, Regterschot et al. investigated the acute effects of passive WBV on executive functions in 133 healthy young adults subjected to six training sessions (frequency 30 Hz, amplitude approximately 0.5 mm) alternating with six rest sessions of two minutes each. The use of the Color–Word Interference Test (CWIT) and Stroop Difference Score (SDS) showed a significant improvement in executive functions, specifically attention and inhibition, in the trained group compared to the control group, suggesting passive WBV as a therapeutic strategy for the elderly and other populations with reduced attention and inhibition, including persons with attention deficit hyperactivity disorder [34].

Importantly, increased neuronal activity would appear to be directly associated with the acute increase in glucose metabolism, as reduced basal glucose levels have been suggested as indicative of brain pathology, as well as being considered an early biomarker for Alzheimer’s disease [35,36]. However, no significant data are available on possible changes in glucose metabolism in the brain after WBV stimulation. In this context, Boerema et al. investigated the impact of a 5-week WBV intervention on brain activity by assessing glucose metabolism in the murine brain and testing executive functioning and memory in elderly subjects without cognitive deficits. Positron emission tomography (PET) scans performed in the mice revealed that glucose uptake was not altered by WBV exposure, although WBV improved cognition and motor performance and reduced arousal-induced home cage activity. Interestingly, cognitive tests in humans showed a selective improvement in the Stroop Color–Word test, known to be positively correlated with cholinergic activity, disconfirming WBV as a safe intervention to improve brain functioning, albeit with variable effects depending on the protocol used [37]. In agreement with this, Alashram and colleagues highlighted in a systematic review that included twenty randomized controlled trials and pseudo-randomized controlled trials how short-term WBV training represents a valid strategy to reduce lower limb spasticity and improve mobility and balance in patients with neurological disorders, although the optimal parameters of an appropriate WBV protocol remain unclear and current evidence is limited by heterogeneity and a scarcity of research [38].

### 3.2. WBV Promotes Musculoskeletal Health

The effects of WBV on the musculoskeletal system have been extensively documented in both animal and human models, suggesting, among the main benefits, an increase in muscle mass and strength and bone mineral density, as well as improved motor performance and general health [39,40]. Not surprisingly, vibratory training is currently used as a valid strategy to prevent various diseases, including sarcopenia, osteoporosis, and arthrosis, as well as to improve musculoskeletal function and joint stability [41,42]. In this context, Matsumoto and Goto investigated the effects of low-intensity WBV in 13-week-old mice undergoing tibial perforation, finding an improvement in bone healing induced by an increase in vascular growth [43]. In agreement with this, Keijser and colleagues observed that exposure to a 5-week WBV protocol (30 Hz, 5 or 30 min per day for 5 weeks) in CD1 mice improved motor performance and object recognition in a dose-dependent manner, confirming the duration of the training session as another key parameter to consider when designing appropriate protocols [44]. Similarly, Cariati et al. observed how a WBV protocol characterized by low frequency, short vibration exposure time and longer recovery period between two consecutive sessions improved musculoskeletal health in a middle-aged mouse model. In contrast, light and electron microscopy analyses showed altered sarcomeric structures, abundant inter-fiber fibrosis and a higher percentage of atrophic fibers when animals were exposed to a more stressful WBV protocol [45]. These results were recently confirmed by the same authors in a study on 4-month-old young mice, showing that animals exposed to the less-stressful WBV protocol had the largest mean muscle fiber diameter and the least amount of inter-fiber connective tissue compared to the other experimental groups. Interestingly, histological and morphometric analysis also showed an improvement in the qualitative characteristics of bone tissue, such as higher bone volume and trabecular thickness and less trabecular separation compared to sedentary animals [32]. Indeed, increased bone mass and improved structural parameters in healthy young rodents are well documented. Specifically, a WBV protocol of 5 days per week for 3 weeks increased femoral cortical thickness, cross-sectional area and femoral trabecular bone cell activity in 7-week-old male mice [46]. In addition, a WBV protocol of 15 min/day increased the rate of bone formation in the endocortical surface of the tibial metaphysis and reduced osteoclastic activity in the tibial trabecular bone in 8-week-old female mice [47]; additionally, an increase in tibial trabecular bone fraction was found in 1-week-old mice with osteogenesis imperfecta [48]. Finally, an increase in bone mass and improvement in structural parameters after WBV exposure were also found in young rodents with spinal cord injury, unloading or oophorectomy, confirming it as a valid strategy in the management of bone disorders [49,50,51].

WBV has been shown to produce osteogenic effects, counteracting age-related changes in bone mass. However, contradictory results have been provided regarding the effects of WBV on bone mass in postmenopausal and elderly women. Ruan et al. found a 4.3% increase in the BMD of the lumbar spine and a 3.2% improvement in BMD of the femoral neck in postmenopausal women with osteoporosis exposed to 6 months of WBV (duration 10 min, 5 times per week, frequency of 30 Hz and amplitude of 5 mm) [52]. In agreement with this, ElDeeb and colleagues recently showed how exposure to WBV twice a week for 24 weeks improved leg muscle work and lumbar and femoral BMD in 43 postmenopausal women with low BMD [53]. In contrast, Slatkovska et al. found no increase in calcaneal BMD in postmenopausal women exposed to vibration training for 12 months (frequency of 90 or 30 Hz, with a peak acceleration of 0.3 g) and treated with calcium and vitamin D supplements [54]. Similarly, Rubin et al. found no changes in the bone mineral content (BMC) of the spine, hip, and distal radius in postmenopausal women after WBV (frequency of 30 Hz and size of 0.2 g) [55]. More recently, Marín-Cascales and colleagues conducted a systematic review and meta-analysis evaluating previously published randomized controlled trials investigating the effects of WBV on total, femoral neck, and lumbar spine BMD in postmenopausal women, in order to identify potential moderating factors explaining the adaptations to this type of exercise. Interestingly, vibratory training has been observed to improve the BMD of the lumbar spine in postmenopausal women, especially in those under the age of 65, confirming it as a potential non-pharmacological intervention to improve bone mass in postmenopausal and elderly women, particularly on the lumbar spine, which has been shown to be the most sensitive area [56].

Finally, the improved performance of the balance bundle, object recognition and increased activity of the cholinergic system in the somatosensory cortex and amygdala of experimental mouse models has directed the use of WBV in the management of patients with neurodegenerative diseases such as Parkinson’s disease (PD), especially in the treatment of motor symptoms such as bradykinesia, tremor, muscle rigidity and postural instability. Indeed, the association between PD and significant sensorimotor deficits suggested WBV as a potential strategy to improve sensorimotor function [57]. In this context, Li et al. recently demonstrated how vibratory training represented a passive and safe clinical intervention for patients with moderate PD, especially in cases of motor impairment or poor balance function, with effects comparable to those of conventional therapy [58].

### 3.3. WBV Favours Pain Relief

WBV, known to reduce pain intensity and improve function and quality of life, is currently used to treat patients with low back pain, which is a major cause of disability and remains a major public health concern in many developed countries [59,60]. Several sources have explained the WBV effects on lower back pain treatment, reporting positive correlations between core muscle inactivity, pain intensity and function in patients with lower back pain. In this regard, vibratory training has been suggested to activate muscle fibres and strengthen core stability muscles, improving back function in lower back pain patients [61,62]. Interestingly, the improvement in muscle activity and strength has been attributed to the enhancement of neural factors, such as recruitment and synchronization, as well as inter- and intramuscular coordination and proprioceptor responses [63]. In this regard, Rittweger et al., in a 6-month follow-up randomized controlled trial, suggested that both lumbar extension and WBV could alleviate pain and improve the associated limitation in everyday life [64], as well as direct evidence of significant effects in patients with chronic lower back pain after a 12-week WBV therapy compared to no treatment was provided [65]. WBV has also been proposed to increase muscle spindle activity and cause a stretch-reflex response of the trunk muscles, thus activating and strengthening the muscles in patients with lower back pain [66]. In fact, a tonic vibration reflex (TVR) has previously been shown to be provoked by direct mechanical vibrations applied to the muscle belly, just as vibration-induced neuromuscular activation leading to TVR would appear to be primarily determined by muscle spindle reflexes [67]. Finally, proprioception deficits in the lumbosacral region often cause dysfunction and spinal instability in lower back pain patients, suggesting WBV as an alternative method to improve proprioceptive function by activating the proprioceptors of lower back pain patients [68,69]. Due to the presence of pain, many pathologies prevent affected individuals from performing exercises on their own, thus hindering rehabilitation. Fortunately, the use of external mechanical vibratory stimuli, which reduces the deficit in voluntary muscle activation by the neuromuscular stretch reflex, has proven to be an effective method for people with severe disabilities [70]. Furthermore, an alteration and attenuation of pain perception has been reported as a consequence of mechanical vibration in large-diameter fiber systems [71]. As reported by Gate in the pain control theory, the interpretation of pain occurs in the higher centers that receive afferent neural signals from the dorsal horn of the spinal cord. Therefore, the synchronous activation of more Aα/Aβ fibers stimulating the dorsal horn of the spinal cord could be responsible for the reduction of pain caused by vibrations [72]. In this context, Sonza et al. recently observed a significant reduction in sensitivity to Aβ fibers mediated by mechanical allodynia and C fibers already mediated by thermal stimulation after the third WBV session in a mouse model of chronic pain. Notably, a strong influence of vibratory training was found on tactile pressure mechanoreceptors, confirming its efficacy in rehabilitation programs based on sensitivity and pain reduction [73].

In summary, the main evidence reported on the effects of WBV on the brain and musculoskeletal system is presented in Table 1.

## 4. Molecular Mediators Involved in WBV Effects

Although the effects of WBV are widely known, the underlying biological mechanisms have yet to be elucidated, as has the identification of a protocol tailored to the individual’s characteristics for a specific personalized intervention [74,75]. However, in recent years, several studies have been conducted in this field, and some key molecules have been proposed to be responsible for the effects of WBV on the whole organism.

In particular, improved cognitive function has been suggested to depend upon increased production of neurotrophins, known mediators of neuronal development, survival, and function [76]. In this context, brain-derived neurotrophic factor (BDNF) appears to be the most susceptible to exercise-induced regulation and may be among the main contributors to the cascade of molecular and cellular events that support brain plasticity. Indeed, BDNF is considered the main neurotrophin linked to neuronal plasticity, playing a key role in neuronal differentiation and survival [77]. Numerous studies have shown a close correlation between increased BDNF levels and aerobic exercise, while it is unclear how mechanical vibrations may influence the expression of this neurotrophin. In this regard, Simão et al. proposed that the combination of vibratory training with squat exercises improves lower limb muscle performance in elderly women with knee osteoarthritis, probably through an increase in plasma BDNF levels, suggesting a role in the modulation of neuromuscular plasticity [78]. Similarly, Ribeiro and colleagues demonstrated that exposure to WBV for 6 weeks promotes an increase in plasma BDNF levels in association with an improvement in lower limb muscle strength, aerobic capacity, clinical symptoms and quality of life in patients with fibromyalgia syndrome [79]. Interestingly, under pathological conditions in rodent models of cerebral ischemia or stroke, WBV has been observed to stimulate the expression of several mediators involved in neurogenesis, including BDNF, insulin-like growth factor (IGF-1) and doublecortin (DCX) [80]. In this regard, Oberste et al. conducted a double-blind randomized controlled trial to study the effects of a 6-week WBV protocol in adolescent patients hospitalized for major depressive disorder. Notably, antidepressant effects of vibratory training were found in association with increased serum levels of BDNF, IGF-1 and inflammatory markers [81].

Irisin, a polypeptide generated by the cleavage of fibronectin type III domain-containing protein 5 (FNDC5), is also undoubtedly involved in physiological adaptations to exercise, as its expression is known to increase abundantly during exercise in musculoskeletal tissue and nerve tissue. Importantly, BDNF-mediated effects in brain tissue could be enhanced by FNDC5, as its exercise-induced up-regulation is known to result in the up-regulation of BDNF [82]. Furthermore, the increased production of irisin through exercise could influence the bone–muscle crosstalk, promoting an increase in the proliferative and mineralizing capacity of osteoblasts [83], as well as promoting muscle growth through a signaling pathway that reduces the expression of myostatin, the main negative regulator of muscle growth [84]. In this regard, Yang and colleagues observed an up-regulation of osteogenic markers, such as osterix, RUNX2 and osteopontin, in MC3T3-E1 murine osteoblasts in response to irisin, suggesting its involvement in bone formation and mineralization processes [85]. Similarly, Shan and colleagues showed that knockout of the myostatin gene produces an increase in the muscle mass and browning of adipose tissue, effects known to be attributed to irisin [86]. In agreement with this, Cariati et al. recently studied the potential effects of WBV on the expression of FNDC5 and tissue-specific markers such as BDNF in brain, myostatin in muscle, and collagen I (COL-1) in the bone of 4-month-old young mice [32]. Interestingly, increased expression of FNDC5, improved tissue structural organization and increased BDNF expression were detected after exposure to a WBV protocol with shorter vibration exposure times and longer recovery times. Furthermore, increased FNDC5 expression was found in the muscles and bones of trained mice, as well as reduced myostatin expression and increased COL-1, confirming WBV as a valid strategy to preserve musculoskeletal health [32].

## 5. WBV vs. Exercise: Do We Have a Chance?

The effectiveness of adding WBV to conventional training generally remains an open question. On the one hand, several sources have reported the combination of WBV and physical therapy as a valid strategy to significantly increase muscle strength and power, flexibility and BMD, as well as to reduce abdominal fat [87,88,89,90]. Specifically, a significant increase in isometric and dynamic knee extensor strength and countermovement jump height was observed in healthy women undergoing 12 weeks of WBV and resistance training [91]. Similarly, Osawa and colleagues observed significant improvements in knee extensor strength and countermovement jump height in young and older adults undergoing WBV combined with routine exercises [92]. Furthermore, Berschin et al. investigated whether WBV could be considered a practical alternative to a standard exercise programme in 40 patients undergoing anterior cruciate ligament (ACL) reconstruction. Notably, exposure to WBV resulted in better neuromuscular performance, in terms of strength and co-ordination, as well as improved postural control in a short period of time, making it a good alternative to a standard exercise programme in ACL rehabilitation [93]. In contrast, Cochrane et al. found no improvement in countermovement jump height, sprint speed and agility performance in non-elite athletes subjected to 9 sessions of WBV training [94]. Similarly, Rogan et al. concluded that adding WBV to physical therapy did not improve muscle strength in healthy elderly people [95], and Anwer and colleagues found no beneficial effects of WBV on quadriceps muscle strength in patients with knee osteoarthritis [96]. More recently, Rasti and colleagues conducted a randomized clinical trial to compare the effects of physical training with and without WBV on flexibility, vertical jump height, agility, and pain in 24 athletes with patellofemoral pain (PFP), a well-known musculoskeletal condition prevalent among active young adults [97]. Interestingly, physical therapy with and without WBV was observed to significantly reduce pain and increase agility, vertical jump height and flexibility in athletes with PFP. However, WBV implementation to routine physical therapy increased the latter’s effects on flexibility, confirming vibratory training as a valuable additional strategy to increase the effectiveness of conventional physical therapy and optimize athletic performance [97]. In agreement with this, Gloeckl et al. demonstrated that balance training performed on a WBV platform induced greater benefits in terms of balance performance and muscle power than conventional training in patients with severe chronic obstructive pulmonary disease (COPD) and functional impairment [98]. Finally, Guadarrama-Molina and colleagues found a significant improvement in functional balance status in 45 PD patients after exposure to conventional therapy and WBV, suggesting their combination as a viable therapeutic alternative to improve quality of life in PD patients compared to conventional therapy alone [99].

## 6. Conclusions

WBV is undoubtedly an expanding area of research with clear potential for medical applications. However, this field could benefit from more standardized and customized protocols. In this regard, the identification of a WBV protocol suitable for a specific age group could pave the way for intervention studies targeting subjects forced to a sedentary lifestyle. Indeed, the future goal of research in this field should be to tailor appropriate training protocols to the individual’s characteristics, as each subject possesses unique and nuanced characteristics at the molecular, physiological, environmental exposure and behavioral levels, thus requiring specific interventions. The use of translational research can facilitate this by considering vibration amplitude, vibration frequency, method of application, session duration/frequency and total duration of intervention as key parameters of WBV. In this regard, studies performed with significantly different protocols in terms of frequency, amplitude, acceleration, and duration were compared in our manuscript, posing difficulties in comparing the evidence analyzed. Notably, such information was not always found to be complete, and this represents a major limitation for both the studies examined and our narrative review. Importantly, van Heuvelen et al. have recently published guidelines for the correct and complete drafting of in vitro and in vivo studies, in animal and human models, relating to vibratory training, indicating all the variables that must necessarily be specified in scientific papers [100].

Nevertheless, the study of physiological adaptations induced by specific WBV protocols is crucial for the development of preventive and/or therapeutic strategies. In this context, WBV training represents a real potential application for improving dose–response effects and counteracting multi-organ decay related to age and/or degenerative diseases, foreseeing an improvement in quality of life and a potential reduction in public health costs. Therefore, substantial investigations into the underlying molecular mechanisms are needed in order to identify the role of potential key mediators involved in physiological adaptations to WBV that could be used as markers of efficacy of the protocol used.

## Figures and Tables

**Table 1:** A schematic representation of the main scientific evidence on the WBV effects.

References	Experimental Groups	Objectives	WBV Parameters	WBV Effects
[29]	15 female Sprague-Dawley rats (9–12 months) exposed to transient middle cerebral artery occlusion: CTRL group: *n* = 8 WBV group: *n* = 7	Testing the efficacy of WBV in counteracting post-ischemic stroke fragility and brain damage in reproductively senescent female rats	Frequency: 40 Hz Acceleration: 0.3× *g* Duration: 15-min series, twice a day for 5 days/week for 30 days	Significant reduction in inflammatory markers and infarct volume Significant increase in BDNF expression Improvement in functional activity
[30]	18 male rats (3 months): CTRL group: n = 5 CTRL group with CRS: n = 6 WBV group with CRS: n = 7	Examining the WBV effects on neuronal loss, synaptic protein and neurotrophin expression in a rat model of chronic restraint stress-induced depression	Frequency: 30 Hz Amplitude: 4.5 mm Duration: 30 min a day, 6 days a week for 8 weeks	Attenuation of depressive behavior, with reduced immobility and improved mood Improvement of spatial memory deficits and reduction of hippocampal neuronal neurodegeneration Preservation of brain morphology Maintenance of BDNF and IGF-1 protein levels
[31]	32 male BALB/c mice 20 mice of 4 months: CTRL group: n = 4 CTRL WBV group: n = 4 WBV groups: n = 12 12 mice of 24 months: CTRL group: n = 4 CTRL WBV group: n = 4 WBV group: n = 4	Assessing the WBV ability to modulate hippocampal synaptic plasticity	Frequency: 90 Hz (A protocol) and 45 Hz (B and C protocol) Amplitude: 1.1 mm at 90 Hz and 1.5 mm at 45 Hz Acceleration: 2× *g* at 90 Hz and 2.8× *g* at 45 Hz Duration: 5 series of 3 min with 1 min recovery between series, 3 times a week for 12 weeks (A and B protocols); 3 series of 2 min 30 s with 2 min 30 s recovery between series, 3 times a week for 12 weeks (C protocol)	In the group of young mice, only the C protocol promoted a preservation of normal synaptic activity In the old mice group, the C protocol induced a significant increase in synaptic plasticity and the disappearance of the epileptic tendency found in the CTRL groups
[32]	20 male BALB/c mice: CTRL group: n = 5 CTRL WBV group: n = 5 WBV groups: n = 10	Investigate WBV-induced brain and musculoskeletal adaptations	Frequency: 45 Hz Amplitude: 1.5 mm Acceleration: 2.8× *g* Duration: 5 series of 3 min with 1 min recovery between series, 3 times a week for 12 weeks (B protocols); 3 series of 2 min 30 s with 2 min 30 s recovery between series, 3 times a week for 12 weeks (C protocol)	Preservation of cerebellar and hippocampal architecture, increased mean diameter of muscle fibres, less interfibral connective tissue, and improved bone morphometric parameters after WBV exposure, especially for the C-trained group Significant increase in BDNF and FNDC5 expression in the cerebellum and hippocampus of the C-trained group Increased expression of FNDC5 in muscle and bone of trained mice, in association with reduced myostatin expression in muscle and increased COL-1 in bone
[34]	133 healthy young adults (20.5 ± 2.2.)	Studying the acute effects of passive WBV vibration on executive functions	Frequency: 30 Hz Amplitude: 0.5 mm Duration: 6 sessions of 2 min alternated with 6 recovery sessions	Short-term positive effect on executive functions of attention and inhibition, with a high level of cognitive functioning
[37]	20 C57Bl/6J mice (15 weeks): CTRL group: n = 10 WBV group: n = 10 34 adults over 40: CTRL group: n = 16 WBV group: n = 18	Assessing the WBV impact on brain activity, arousal-induced activity, and executive functioning	For the animal model: Frequency: 30 Hz Amplitude: 0.05 mm Acceleration: 0.098× *g* Duration: 10 min per day, 5 days per week, for a period of 5 weeks For humans: Frequency: 30 Hz Amplitude: 0.5–1 mm Acceleration: 0.9–1.8× *g* Duration: 4 min per day, 4 days per week, for a period of 5 weeks	Significant increase in performance in the equilibrium beam crossing test and reduction of excitation-induced activity in animals exposed to WBV Improvement of executive functions of attention and inhibition assessed by Stroop Color-Word test in men exposed to WBV
[43]	48 male C57BL/6 mice (13 weeks) with cortical perforation on the tibial bone: CTRL group: n = 24 WBV group: n = 24	Investigating the WBV effects on vascularization during the early stages of fracture healing	Frequency: 30 Hz Acceleration: 0.1× *g* Duration: 20 min daily for 12 days	Modulation of vascularization and acceleration of regeneration in terms of volume and bone mineral density
[44]	44 male CD1 mice (3 months): CTRL groups: n = 22 WBV groups: n = 22	Assessing the WBV effects on attention and motor performance	Frequency: 30 Hz Acceleration: 1.9× *g* Duration: 5 or 30 min a day, 5 days a week for 5 weeks	Significant improvement in balance beam performance and object recognition only after exposure to a 5-min WBV protocol
[45]	20 male BALB/c mice (12 months): CTRL group: n = 4 CTRL WBV group: n = 4 WBV groups: n = 12	Studying muscle adaptations to WBV in a middle-aged mouse model	Frequency: 90 Hz (A protocol) and 45 Hz (B and C protocol) Amplitude: 1.1 mm at 90 Hz and 1.5 mm at 45 Hz Acceleration: 2× *g* at 90 Hz and 2.8× *g* at 45 Hz Duration: 5 series of 3 min with 1 min recovery between series, 3 times a week for 12 weeks (A and B protocols); 3 series of 2 min 30 s with 2 min 30 s recovery between series, 3 times a week for 12 weeks (C protocol)	Improved muscle structure, with well-preserved mitochondria, correct sarcomeric organization and increased fibre diameter only in groups trained with B and C protocols
[46]	58 male mice (7 weeks): CTRL groups: n = 34 WBV groups: n = 24	Identifying a WBV protocol that promotes bone growth in healthy young mice	Frequency: 90 Hz Acceleration: 0.5–2× *g* Duration: 15 min a day for 5 days a week for 3 to 9 weeks	Increased thickness of the femoral cortical area, together with reduced expression of sclerostin and DMP1 in cortical osteocytes, only after WBV exposure for 3 weeks
[47]	38 female BALB/cBxJ mice (8 weeks): CTRL groups: n = 18 WBV group: n = 10 WBV-R group: n = 10	Investigating the influence of WBV on trabecular and cortical formation and resorption processes in the growing skeleton	Frequency: 45 Hz Acceleration: 0.3× *g* Duration: 15 min a day for 5 days a week for 3 weeks. In the WBV-R group, each training session was interspersed with a 10-s recovery period	Reduction of osteoclastic activity in the trabecular metaphysis and tibial epiphysis, by 33% and 31% respectively, after WBV exposure No significant changes at the cellular level were observed in the WBV-R group
[48]	24 homozygous wild type (B6C3Fe-a/a-+/+) female mice 24 homozygous oim (B6C3Fe-a/a-oim/oim) female mice	Studying the WBV effects on cortical and trabecular bone formation in young mice with osteogenesis imperfecta	Frequency: 45 Hz Acceleration: ±0.3× *g* Duration: 15 min a day for 5 days a week for 3 to 9 weeks	Improvement of bone mechanical properties and morphology of trabecular and cortical bone in hind limbs in the osteogenesis imperfecta group
[52]	94 postmenopausal women with osteoporosis: CTRL group: n = 44 (63.73 ± 5.45) WBV group: n = 51 (61.23 ± 8.20)	Investigating the WBV effects on menopause-induced BMD decline and chronic back pain	Frequency: 30 Hz Amplitude: 5 mm Duration: 10 min a day, 5 days a week for 6 months	Increased lumbar and femoral neck BMD and significantly reduced back pain intensity
[53]	43 postmenopausal women: CTRL group: n = 21 (57.29 ± 4.44) WBV group: n = 22 (55.08 ± 4.19)	Studying the WBV impact on muscle work and BMD of the lumbar vertebrae and femur	Frequency: 20–35 Hz Amplitude: 2.5–5 mm Duration: 5 to 10 min, twice a week for 24 weeks	Significant improvement in leg muscle contraction Significant increase in BMD of the lumbar vertebrae and femur
[54]	202 postmenopausal women: CTRL group: n = 67 (60.8 ± 5.5) WBV groups: n = 135 (59.6 ± 6.0–60.5 ± 7.0)	Examining the WBV effects on calcaneal QUS measurements	Frequency: 30 or 90 Hz Acceleration: 0.3× *g* Duration: 20 consecutive min per day for 12 months	No significant improvement on calcaneal QUS measurements after exposure to WBV
[55]	64 postmenopausal women: Placebo group: n = 32 (47–64 years) WBV group: n = 32 (52–64 years)	Investigating the ability of WBV to inhibit bone loss	Frequency: 30 Hz Acceleration: 0.2× *g* Duration: 2 sessions of 10 min a day, 7 days a week for 1 year	Reduced bone loss and increased BMD of the femoral neck and lumbar spine
[58]	29 patients with moderate PD: CTRL group: n = 16 (60.06 ± 3.38) WBV group: n = 13 (61.15 ± 3.72)	Studying the short-term effect of WBV on motor proprioceptive functions in patients with moderate PD	Frequency: 6 Hz Amplitude: 3 mm Duration: 2 treatment sessions consisting of 5 series of 1 min with 1 min of recovery	Significant improvement in motor function
[64]	50 patients with chronic low back pain: LEX group: n = 25 (49.8 ± 6.6) WBV group: n = 25 (54.1 ± 3.4)	Comparing the WBV effects on chronic back pain versus LEX	Frequency: 18 Hz Amplitude: 6 mm Duration: 4–7 min, 2 series per week during the first 6 weeks and 1 series per week for the next 6 weeks	Significant reduction in pain sensation and related disability in both trained groups
[65]	49 patients with non-specific low back pain: CTRL group: n = 24 (59.53 ± 5.47) WBV group: n = 25 (58.71 ± 4.59)	Testing the effectiveness of WBV in counteracting chronic non-specific low back pain	Frequency: 20 Hz Duration: twice a week for 12 weeks, with a recovery day between sessions. The WBV exposure time was 60 s for the first 2 weeks and increased by a further 60 s every 2 weeks	Reduction in functional disability and back pain, with significant improvement in quality of life
[73]	60 male Wistar rats (3 months) CTRL group: n = 12 CTRL group with pain: n = 12 Low-intensity training group: n = 12 WBV group: n = 12 Combined training group: n = 12	Studying the WBV effects in a chronic pain model	Frequency: 42 Hz Amplitude: 2 mm Acceleration: 7.1× *g* Duration: 5 min on the first 5 days and 10 min on the remaining 5 days	Reduced mechanical and thermal sensitivity and hyperalgesia after WBV exposure

WBV: whole body vibration; CTRL: control; BDNF: brain-derived neurotrophic factor; CRS: chronic restraint stress; IGF-1: insulin-like growth factor 1; FNDC5: fibronectin type III domain-containing protein 5; COL-1: collagen I; DMP1: dentin matrix acidic phosphoprotein 1; BMD: bone mineral density; QUS: quantitative ultrasound; PD: Parkinson’s disease; LEX: lumbar extension exercise.

## Data Availability

No new data were created or analyzed in this study. Data sharing is not applicable to this article.
